# Recognizing chemicals in patents: a comparative analysis

**DOI:** 10.1186/s13321-016-0172-0

**Published:** 2016-10-28

**Authors:** Maryam Habibi, David Luis Wiegandt, Florian Schmedding, Ulf Leser

**Affiliations:** 1 Knowledge Management in Bioinformatics, Humboldt-Universität zu Berlin, 12489 Berlin, Germany; 2 Averbis GmbH, 79106 Freiburg, Germany

**Keywords:** Chemical named entity recognition, Patent mining, Ensemble approach, Simple chemical elements, Performance measurements

## Abstract

Recently, methods for Chemical Named Entity Recognition (NER) have gained substantial interest, driven by the need for automatically analyzing todays ever growing collections of biomedical text. Chemical NER for patents is particularly essential due to the high economic importance of pharmaceutical findings. However, NER on patents has essentially been neglected by the research community for long, mostly because of the lack of enough annotated corpora. A recent international competition specifically targeted this task, but evaluated tools only on gold standard patent abstracts instead of full patents; furthermore, results from such competitions are often difficult to extrapolate to real-life settings due to the relatively high homogeneity of training and test data. Here, we evaluate the two state-of-the-art chemical NER tools, tmChem and ChemSpot, on four different annotated patent corpora, two of which consist of full texts. We study the overall performance of the tools, compare their results at the instance level, report on high-recall and high-precision ensembles, and perform cross-corpus and intra-corpus evaluations. Our findings indicate that full patents are considerably harder to analyze than patent abstracts and clearly confirm the common wisdom that using the same text genre (patent vs. scientific) and text type (abstract vs. full text) for training and testing is a pre-requisite for achieving high quality text mining results.

## Background

Patents are an economically important type of text directly related to the commercial exploitation of research results. They are particularly essential for the pharmaceutical industry, where novel findings, such as new therapeutics or medicinal procedures, result from extremely cost-intensive, long-running research projects, but often are relatively easy to copy or reproduce [1]. Accordingly, a large number of commercial services exists regarding the formulation and retrieval of patents [2], and large companies devote entire departments to the creation, the licensing, and the defense of their patent portfolio. Such services must be supported by proper computational tools, as the number of patents is increasing rapidly. For instance, the European Patent Office granted 614,850 patents since 2006 [3]; the size of the United States Patent and Trademark Office corpus currently is 6,718,054 patents with a yearly increase of roughly 300,000 over the last 5 years [4]. However, current tools for patent management mostly support keyword search [5–9], whereas only few projects exist that target the extraction of specific facts from patents [10, 11].

In this work, we study the identification and extraction of chemical names^1^ from patents. By extraction, we mean the identification of left and right borders of mentions in patents, a task usually referred to as Named Entity Recognition (NER). Extracting chemicals from scientific articles has been a topic of ample research over the last 15 years, leading to the creation of high quality tools like OSCAR [12] or ChemSpot [13] which focus on the particularities of chemical names when compared to other entities, such as genes or species [13–16]. However, the extraction of chemicals from patents has been neglected by the research community for long, mostly due to the difficulties in obtaining computer-readable patents at large—compared to the simple procedures necessary to download scientific articles from sources like PubMedCentral^2^—and the lack of properly annotated patents, i.e. gold standard corpora. It is tempting to apply tools and models developed for scientific articles on patents, but patent texts are quite different from scientific articles. They are typically much longer, yet have a lower word density [17]. Their writing is more difficult to understand as the protection of broad claims and a mild obfuscation of procedures are established means to increase patent value and decrease the likelihood of being reproduced [5]. Therefore, it is rather unclear whether tools developed for scientific articles perform equally well on patent data.

Since 2012, two gold standard full-text patent corpora have been published: the chapati corpus [18] and the corpus from the BioSemantics research group [19]. The field was further boosted by a recent international competition, the CEMP task at BioCreative V. For this task, two large corpora for training and development were prepared and used by 21 teams to develop patent-specific solutions [20–22], achieving the F-measure values of up to 89% (87% precision and 91% recall) using an ensemble approach. However, both corpora consist only of patent titles and abstracts, while commercially interesting applications critically depend on analyzing full texts, as a significant number of entities is not even mentioned in an abstract [17]. Furthermore, international challenges are important to make different approaches comparable and also provide a strong incentive for groups to enter a field [11], yet their performance results are difficult to extrapolate due to the relatively high homogeneity of training and test data within the competition. In contrast, real applications typically have to perform information extraction on diverse text collections without having accordingly diverse training data. To mimic such situations, cross-corpus evaluations can be used, where the performance of a tool trained on one corpus is measured on another corpus following different annotation guidelines [23].

In this paper, we take this idea one step further and perform a cross-text-genre evaluation by assessing the performance of chemical NER tools trained on scientific articles—a problem much better researched—on patent corpora. We choose tmChem [24] and ChemSpot [13], two state-of-the-art tools for chemical NER from scientific articles, and evaluate their performance (without retraining) on all four freely available gold standard patent corpora with annotations of chemical mentions. We put emphasis on the differences between evaluations on abstracts versus full texts, showing that the latter is a considerably harder task for current tools. We also compare results on the instance level, showing that, despite having similar performance numbers, tmChem and ChemSpot actually return quite different results. This makes the creation of ensembles attractive, two of which we evaluate on all four corpora. We also contrast our cross-text-genre results to those obtained after retraining a chemical NER tool on patent corpora, showing that taking away the text-genre difference significantly boosts results, i.e., that the different characteristics of patent versus scientific texts strongly impact chemical NER performance. Overall, our results emphasize the common wisdom that using the same text genre (patents vs. scientific articles) and text type (abstracts vs. full texts) for training and application is a pre-requisite for achieving high-quality text mining results.

## Methods

In this section, we describe the characteristics of the four freely available gold standard patent corpora and present the two chemical NER systems utilized in our study. We also explain two ensemble approaches, our evaluation metrics, and the text preprocessing techniques employed.

### Patent corpora

We use all four currently existing gold standard patent corpora with annotations for chemicals. Two of them contain only the title and the abstract of patents, while the other two use complete patent documents. The two abstract corpora, denoted as CEMP_T and CEMP_D, contain 7000 patents each. They were originally developed for training and development purposes within the CEMP (chemical entity mention in patents) task [11] of the BioCreative^3^ V challenge. The chapati corpus [18] is the result of a collaboration between the European Patent Office^4^ and the CHEBI^5^ team. Chemical entities were manually annotated for 40 complete patent documents and normalized to CHEBI identifiers. The fourth corpus, noted here BioS, was prepared by the BioSemantics^6^ research group and covers 200 full patent documents [19]. For this corpus, patents were automatically pre-annotated and then manually curated by at least one annotator group consisting of two to ten annotators. Table 1 shows statistics on these corpora like corpus size (the number of tokens separated by space), number of documents, classes of annotated chemicals and number of annotated entities.

**Table 1:** The details of the gold standard patent corpora containing the annotations for chemicals

Corpus	Number of patents	Annotated entities	Number of annotations
CEMP training set (CEMP_T) [11, 25] $\approx$660 thousand token	7000 patents (title and abstract)	ABBREVIATION, FAMILY, FORMULA, TRIVIAL, MULTIPLE, SYSTEMATIC, IDENTIFIERS	33543 (without normalization)
CEMP development set (CEMP_D) [11, 25] $\approx$650 thousand token	7000 patents (title and abstract)	ABBREVIATION, FAMILY, FORMULA, TRIVIAL, MULTIPLE, SYSTEMATIC, IDENTIFIERS	32142 (without normalization)
CHEBI patent corpus (chapati) [18] $\approx$265 thousand token	40 full patents (title, abstract, claims, description)	CLASS, CHEMICAL, ONT, FORMULA, LIGAND, CM	18746 (normalized to CHEBI identifiers)
BioSemantic patent corpus (BioS) [19] 11,500 pages and $\approx$4.2 million token	200 full patents (title, abstract, claims, description)	IUPAC, SMILES, InChi, ABBREVIATION, MOA, DISEASE, FORMULA, REGISTRY NUMBER, GENERIC, TRADEMARK, CAS NUMBER, TARGET	400125 (without normalization)

Note that these corpora were annotated using different annotation guidelines. The corpora from the CEMP task and BioSemantics group were annotated using two specific annotation guidelines, while the chapati curators considered all entities that could be automatically mapped to a CHEBI identifier. The annotation guidelines vary in several aspects. For instance, the IUPAC name “water” should not be annotated as a chemical in the CEMP corpora but it should be in the BioS corpus [19]. Additionally, simple chemical elements are annotated in the CEMP corpora but not in the BioS corpus.

Additionally, we compare our results on the patent corpora with those achieved on two corpora consisting of scientific articles: CHEMDNER and CRAFT. The CHEMDNER corpus [25] was developed for the CHEMDNER task at the BioCreative IV challenge. The corpus consists of scientific abstracts that were annotated using the same annotation guideline used for the CEMP task. In this work, we only used the test set, containing 3000 abstracts, since the training set and the development set were used for training tmChem. The CRAFT corpus [26] consists of 97 scientific full texts, yet only 67 of these have been publicly released to date. The chemical annotations of the CRAFT corpus are limited to terms from the CHEBI database.

For illustration (see “Text-genre statistics” section), we briefly analyzed differences between patents and scientific articles in terms of the average number of words per sentence (sentence length), the average number of words per document (document length), the average number of unique/non-unique TLAs^7^ in a document, and the average number of figures and tables per document. To this end, we used a collection of randomly selected full patent documents from European Patent Office^8^, and a set of randomly selected full journal articles from PubMedCentral. All texts were from year 2015; patents were selected after classification to ensure biomedical topics. We calculated the number of tables and figures by counting the number of their tags in xml format.

### Chemical NER systems

Over the last years, many tools have been presented for chemical NER, including tmChem [24], ChER [10], ChemSpot [13], becas [27], OSCAR [12] or ChemXSeer-tagger [28]. We chose two of them based on their good overall performance in a number of evaluations: (1) tmChem developed by Leaman et al. [24], as the best system at the CHEMDNER challenge in BioCreative IV^9^ [16], (2) ChemSpot^10^ which was introduced in the year 2012 and outperformed all other tools for many years. Table 2 gives an overview of the two tools.

**Table 2:** Details on the chemical NER tools in terms of training sets, databases to which the entities are normalized, classes of chemicals addressed, and tokenization methods

NER tool	Training set	Databases	Classes	Tokenization method
tmChem [24]	CHEMDNER corpus at BioCreative IV (training and development sets)	CHEBI	SYSTEMATIC	Tokenization at every non-letter and non-digit characters, number- letter changes and lower case letter followed by an uppercase letter
		MESH	FORMULA	
			FAMILY	
			TRIVIAL	
			IDENTIFIER	
			MULTIPLE	
			ABBREVIATION	
ChemSpot [13]	A subset of SCAI Corpus [29] containing only IUPAC	ChemIDplus	SYSTEMATIC	Tokenization at every non-letter and non-digit characters and number-letter changes
		CHEBI	FORMULA	
		CAS	FAMILY	
		NUMBER	TRIVIAL	
		PubChem	IDENTIFIER	
		InChI	MULTIPLE	
		DrugBank	ABBREVIATION	
		KEGG		
		Human		
		Metabolome		
		MESH		

ChemSpot employs a hybrid approach, in which the results of a CRF model trained to recognize IUPAC entities are combined with dictionary matching to find other chemical names. TmChem uses ensembles of two CRF models, called Model1 and Model2, with different setups and configurations. As the implementation of the ensembles were not freely avaliable, we performed our experiments using the individual models. We limited our study to the results from Model1, noted as tmChem, as its performance was always very close to or higher than that of the Model2 [22, 24]. Both tmChem and ChemSpot build on BANNER as CRF implementation [30], but use different feature sets, tokenization methods, and training sets. Both tools were trained on scientific abstracts, but the training corpora comprise different articles and were annotated based on different annotation guidelines [31]. Note that the annotation guideline used to annotate the training set of tmChem is very similar to the one used to annotate the two CEMP patent corpora. Both tools normalize extracted entities. TmChem maps the entities to identifiers from CHEBI and MESH, whereas ChemSpot maps them to further databases, like InChI and DrugBank.

### Ensemble NER systems

A comparison of the concrete set of entities returned by tmChem and ChemSpot (see “Comparison at instance level” section), respectively, showed significant divergence. Since ensembles of NER tools often outperform individual tools [28], we also measure the performance of two ensembles produced by merging the results of tmChem and ChemSpot. One ensemble system, called Ensemble-I, accepts a mention as a chemical name when both tmChem and ChemSpot recognize it as such. The second ensemble, noted Ensemble-U, considers a span as a chemical name when it is recognized by at least one of the two systems.

### Evaluation metrics

Performance values were computed in terms of precision, recall, F-measure, and true positive (TP), false positive (FP), and false negative (FN) counts; in all cases, only exact span matches were considered. Precision measures the ratio of correctly predicted chemical entities to all predicted entities; recall is defined as the ratio of correctly predicted entities to all annotated entities within a corpus. F-measure is the harmonic mean of the precision and the recall values.

We measured performance values using the conlleval^11^ script run over the prediction and reference annotation files in IOB format. We also compared the different methods with respect to the execution time on patents and scientific articles.

### Text preprocessing

The different gold standard corpora were available in different text formats which we homogenized before running the NER tools. In a first step, each document was converted to plain text format and stored in a single file. Then we transformed these files to the input format defined by each NER tool. For evaluation purposes, we tokenized each prediction and gold standard annotation files as suggested by Klinger et al. [32]^12^, and represented each token in IOB format. We used the Stanford parser^13^ [33] to split the text into sentences and to parse a sentence.

## Results

We first provide evaluation scores for the models trained on the abstract of scientific articles when applied to patents. Then we present an analysis of entities frequently recognized incorrectly as chemicals or non-chemicals by the two systems. Afterwards, we describe results of the two ensemble systems. Finally we present the results of cross-corpus and intra-corpus evaluations to study the impact of the use of different text genres and text types as training and test sets in patent mining.

### Cross-genre evaluation

**Fig. 1:**
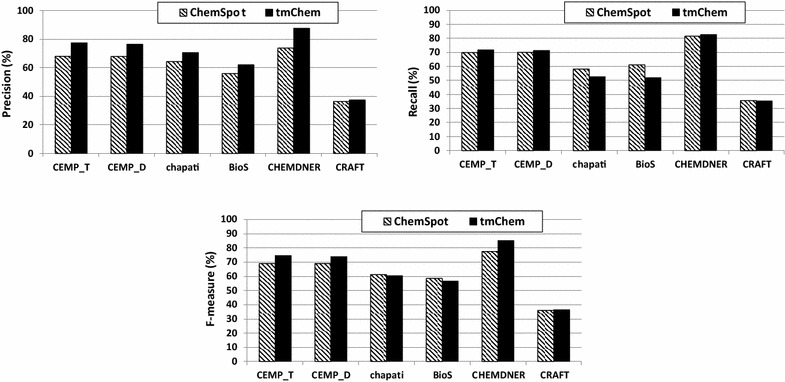
Evaluation scores in terms of precision, recall and F-measure values are measured for ChemSpot and tmChem NER tools over gold standard corpora

We compared the performance of tmChem and ChemSpot on four patent corpora and two corpora consisting of scientific articles in terms of precision, recall, and F-measure. The results are shown in Fig. 1. The following ranking of corpora regarding their assessability by the tools can be inferred: CRAFT<BioS<chapati< CEMPs<CHEMDNER.

We first observe that the performances of both tools are much lower on the CRAFT corpus than on the other corpora. The reason for this discrepancy seems to be that the version of the CHEBI database used for annotating the CRAFT corpus is quite different from that used for training both NER tools [25]. On the CHEMDNER corpus, both tools, despite the use of different training data, have higher performance values than on the other corpora, indicating that they perform best on scientific abstracts—the type of texts they also were trained on. This observation indicates that models trained on scientific abstracts are not quite as capable of recognizing chemical entities from patents. Drawing a conclusion regarding the difference between scientific abstracts and full texts, however, is difficult due to the quite different scope of chemical annotations in the CRAFT corpus [25].

The results, obtained from both tools, also report a ~10% higher performance on patent abstracts compared to full patents, indicating that there is more similarity between patent abstracts and scientific abstracts than between scientific abstracts and full patents.

TmChem has higher F-measure values than ChemSpot (at least 5%) on the CHEMDNER and the CEMPs corpora, both of which follow a annotation guideline similar to that of the tmChem training set. In contrast, ChemSpot was trained on a corpus with a different annotation guideline. However, the F-measure values of both tmChem and ChemSpot were very close on full patent corpora, annotated using guidelines which differ from those of the systems’ training sets. This means that the improvement obtained by tmChem on patent abstracts is likely due to the similarity of the annotation guidelines and not to the superiority of the method. Thus, we cannot conclude which tool is better suited for chemical NER on patents.

### Comparison at instance level

We studied the top 10 entities most frequently incorrectly recognized (FPs and FNs) by tmChem and ChemSpot, respectively. These entities along with their FP and FN counts are shown in Tables 3 and 4. Mistakes made frequently by both tools are in italic font.

**Table 3:** The top 10 entities with highest FP for each chemical NER tool on the four different corpora

CEMP_T		CEMP_D	
ChemSpot	tmChem	ChemSpot	tmChem
Water 951	*Sodium* 128	Water 842	*Sodium* 117
*Alkyl* 260	Sugar 66	*Alkyl* 194	*Nucleotide* 74
*Sodium* 186	CH$_2$ 56	*Sodium* 194	*Ester* 49
DEG 155	*Sulfate* 43	Peptide 153	*Calcium* 49
Peptide 107	NO 42	Chitosan 130	O 46
Chitosan 91	Solvate 40	DEG 108	NO 45
Starch 81	*Alkyl* 39	Parkinson 80	N 44
*Calcium* 74	Hydrogen 38	*Calcium* 76	*Alkyl* 37
*Sulfate* 66	*Calcium* 35	*Nucleotide* 72	Sulfate 37
Parkinson 60	Beta-cyclodextrin 34	*Ester* 67	Beta-cyclodextrin 36

Chapati		BioS	
ChemSpot	tmChem	ChemSpot	tmChem
Factor H 121	CO 127	*Hydrogen* 6246	*Hydrogen* 6179
*Serine* 108	*Serine* 108	*1H* 5034	*Carbon* 5518
Alkyl 81	N 88	*Carbon* 5004	H 3091
*Amino acid* 66	NH–SO$_2$ 64	*3H* 3928	*1H* 2785
SO$_2$–NR<21>R<22 62	*NH–CO–R*<*21* 63	Alkyl 3812	*3H* 2643
CO–R<23 60	*Amino acid* 61	*2H* 2946	*Nitrogen* 2619
*NH–CO–R*<*21* 55	Carbon 57	*Nitrogen* 2878	*Silica* 1466
Ci-I0 54	*Nitroxide* 52	*Silica* 2011	CDCl3 1320
CO–NR<21>R<22 53	C 51	DMSO-d6 1652	*2H* 1259
*Nitroxide* 52	H 46	*Oxygen* 1203	*Oxygen* 1110

Common mistakes are shown in italic

**Table 4:** The top 10 entities with highest FN for each chemical NER tool on the four different corpora

CEMP_T		CEMP_D	
ChemSpot	tmChem	ChemSpot	tmChem
*H* 227	*Alkyl* 226	*H* 233	*Alkyl* 246
*Aryl* 170	*Aryl* 179	*Aryl* 174	*Aryl* 183
*C1-6 alkyl* 115	*H* 173	*Heterocyclic* 133	*H* 179
Heteroaryl 82	*C1-6 alkyl* 121	Heteroaryl 87	*Heterocyclic* 135
*Alkyl* 74	S 86	*N* 76	S 86
N 71	Cyano 85	*C1-6 alkyl* 69	*C1-6 alkyl* 76
Alkoxy 67	*Heterocyclic* 62	Alkoxy 63	*Cyano* 71
*Cyano* 62	*Halo* 55	*Alkyl* 59	*N* 56
*Heterocyclic* 61	Oligonucleotides 50	*Aromatic* 51	Halo 52
*Halo* 51	Opioid 50	*Cyano* 45	*Aromatic* 51

Chapati		BioS	
ChemSpot	tmChem	ChemSpot	tmChem
*Drug* 234	Water 264	*Alkyl* 5295	*Alkyl* 8145
*Ci-I0 alkyl* 160	*Drug* 234	*Aryl* 4698	Water 7142
*NR* 139	Peptide 205	*DMSO* 3184	*Aryl* 5426
*Insulin* 107	*Ci-I0 alkyl* 160	Heteroaryl 2435	Ph 1995
*Aptamer* 92	*NR* 139	Alkoxy 1993	*H* 1921
*Polypeptide* 88	*Insulin* 107	*H* 1869	*Brine* 1822
*SO2R* 65	CN 94	*Brine* 1777	*DMSO* 1490
NH–CO–R 63	*Aptamer* 92	*Inhibitors* 1468	Ethyl acetate 1473
SO$_2$–NR 63	*Polypeptide* 89	*Substituted* 1447	*Inhibitors* 1472
NH–SO$_2$–R 62	*SO2R* 65	Lower alkyl 1422	*Substituted* 1447

Common mistakes are shown in italic

Counting the number of common errors, we find around 50% overlap between the top-10 entities with highest FP counts and nearly 70% overlap between entities with highest FN values between the tools. However, the individual error frequencies are very different. For example, the number of times that the entity “alkyl” is incorrectly recognized as a chemical entity by ChemSpot is around 6 times higher than that of tmChem (in patent abstracts). There are several similar cases in the corpora containing full patents. Similarly, there are also a number of common entities with diverging FN values. By excluding common entities with highly different frequencies, the overlaps between these two tools for patent corpora are reduced to around 40 (for FP counts) and 50% (for FN counts), which indicates the two tools perform rather differently.

#### Error distribution

**Fig. 2:**
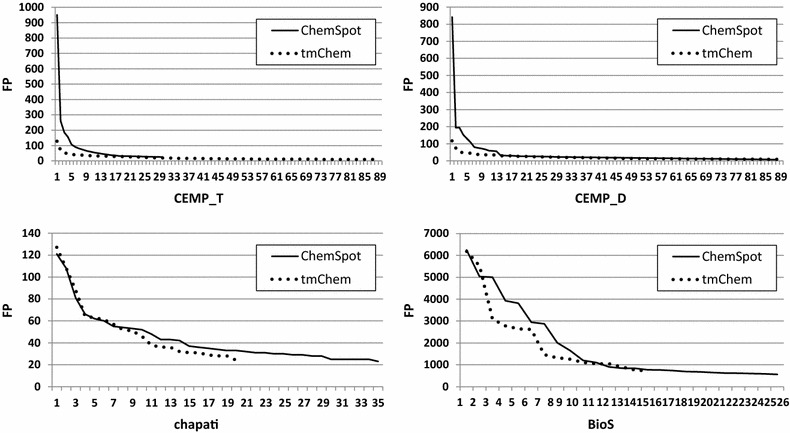
Distributions of FP counts from high to low, for unique entities covering 25% of cases, obtained by tmChem and ChemSpot over all corpora. The *x-axis* represents the number of unique entities. The distributions are notably different for full patents compared to patent abstracts

**Fig. 3:**
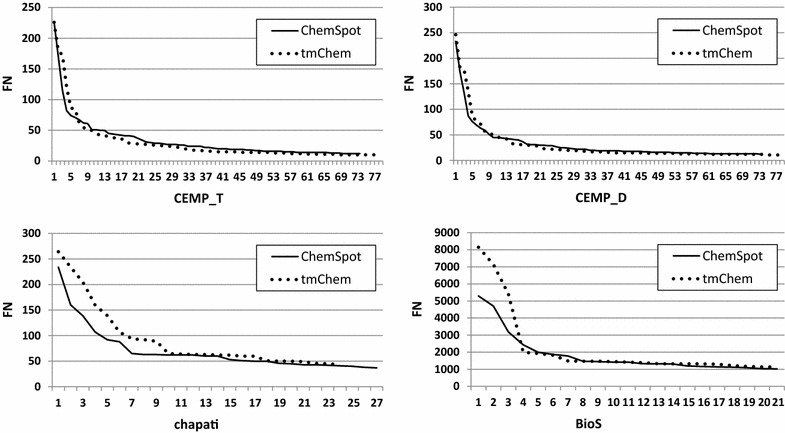
Distributions of FN counts from high to low, for unique entities covering 25% of cases, obtained by tmChem and ChemSpot over all corpora. The *x-axis* represents the number of unique entities. The distributions are very similar for full patents and patent abstracts

We observed many common entities on the lists of errors arisen from tmChem and ChemSpot in Tables 3 and 4, but having highly different frequencies. This observation motivated us to compare patent full texts and abstracts in terms of the distributions of the FP and FN counts measured for unique entities. We depicted the distributions of these values which were sorted from high to low, and covered 25% of error cases, in Figs. 2 and 3.

The distributions over patent abstracts showed that 25% of FP counts are produced by around 90 unique entities for tmChem, and only 30 unique ones for ChemSpot. Moreover, the shapes of the distributions were quite different. Similarly, by comparing the distributions of FP counts over full patents, we observed that the number of unique entities leading to 25% of FPs for tmChem is around 20 and for ChemSpot, it is nearly 30. The shapes of tmChem and ChemSpot distributions were very similar over full patents, but they were different from the ones obtained for ChemSpot over patent abstracts. These results confirm that the distributions of FP counts are different over full patents and patent abstracts.

On the contrary, the shapes of the distributions drawn for FN counts were very similar for both systems over all corpora, but the number of unique entities leading to 25% of FNs over full patents is around 25 while it is 75 for abstracts.

#### Impact of simple chemical elements

Interestingly, there are quite a number of simple chemical elements in these lists (e.g. H, N, S). The appearance of simple chemical elements in both lists of errors indicates that these entities are generally ambiguous and difficult to be correctly predicted, although they are rather irrelevant for many applications in areas such as cell biology or omics studies. This observation encouraged us to study the impact of simple chemical elements on the performance values of different types of patent texts.

**Fig. 4:**
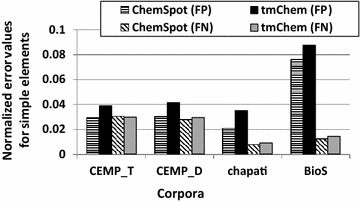
The FP and FN counts of simple chemical elements normalized by the FP and FN counts obtained for the entire entities by tmChem and ChemSpot over all corpora

 First, we computed the FP and FN counts for simple chemical elements normalized with the FP and FN counts of all entities extracted by each NER tool from each corpus as shown in Fig. 4. The results obtained by the two tools demonstrate that the normalized FP values of simple elements are higher than those of FN values for full patents, while they are approximately analogous for patent abstracts. It implies that simple chemical elements are frequently recognized incorrectly as chemicals on full patents.

**Fig. 5:**
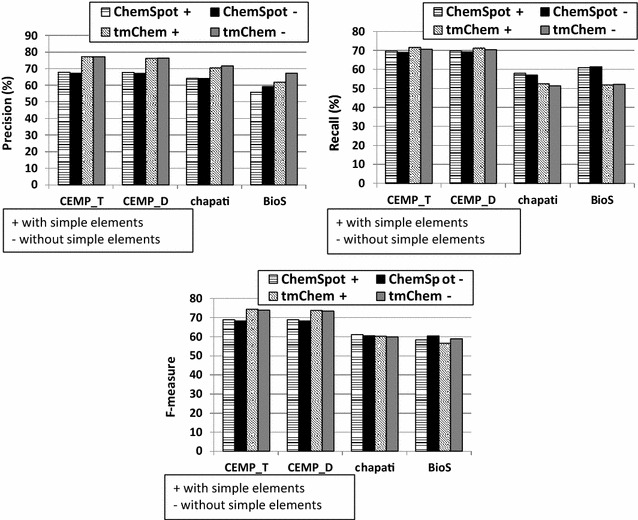
Evaluation scores with regard to precision, recall and F-measure over recognized spans obtained by ChemSpot and tmChem NER tools over gold standard corpora. The results are provided by considering simple elements represented by “+” and without them noted by “−”

Following this observation, we recalculated the performance values, i.e., precision, recall, and F-measure, by excluding the annotations of all simple chemical elements^14^ from the gold standard corpora, and also filtering the simple elements predicted by ChemSpot and tmChem. The results in Fig. 5 show that the performance values of the corpora with the same text type converge into highly close values after removing simple chemical elements. However the impact the removal of simple elements has on the precision values is insignificant, except for the BioS corpus, on which the precision improves by up to 5%. Recall and F-measure values are not strongly affected by ignoring simple chemical elements.

### Ensemble performance

As we found substantial differences in the concrete results computed by tmChem and ChemSpot, we decided to measure the performance of the two ensemble systems obtained by (a) intersecting (Ensemble-I) and (b) unifying (Ensemble-U) the results of the two systems (see “Ensemble NER systems” section).

**Fig. 6:**
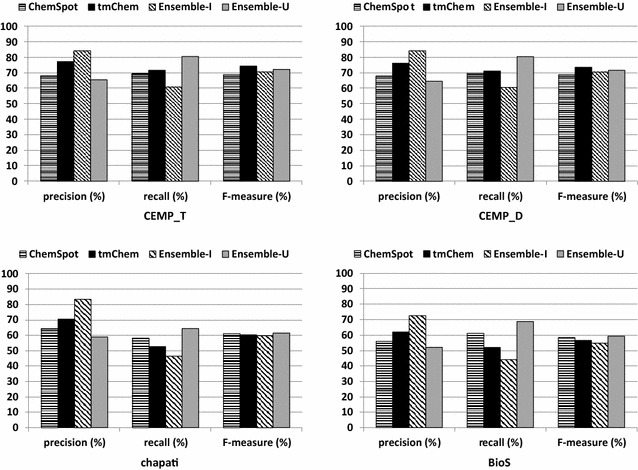
Evaluation scores with regard to precision, recall, and F-measure values over recognized spans obtained by ChemSpot, tmChem, the area of their intersection and union over gold standard corpora

We provide precision, recall, and F-measure values calculated for tmChem, ChemSpot, and for the intersection and the union of their outputs in Fig. 6. As expected, on all corpora, the highest precision is obtained by intersecting the results of the two tools, while the highest recall is provided by unifying the results of the two systems. The results also show that the Ensemble-U provides the highest F-measure value on full patents, while tmChem has the highest F-measure scores on patent abstracts. This can probably be attributed to the use of similar annotation guidelines for training and test sets.

### Cross-text-genre to cross-corpus evaluation

**Fig. 7:**
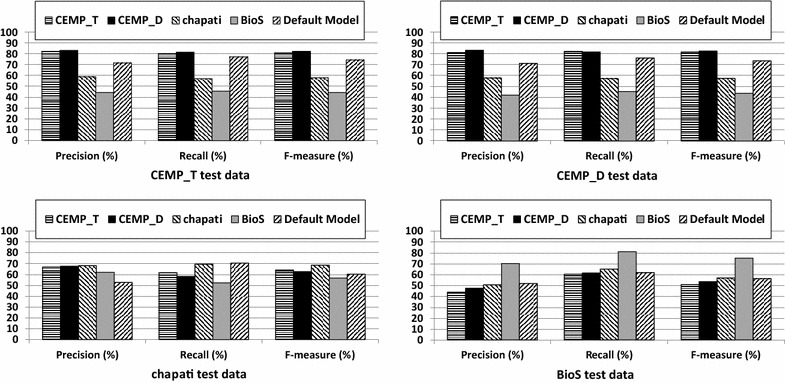
Precision, recall and F-measure values of the models trained using different corpora on the CEMPs, chapati, and BioS patent corpora

We measured the performances of different models obtained by retraining tmChem using patent corpora. We retrained only tmChem because of the well documented process in its public API. We first performed a cross-corpus evaluation, where tmChem was trained on one corpus and evaluated on other corpora. In addition to cross-corpus evaluation, we performed intra-corpus evaluation by assessing the performances of the models using fourfold cross validation. The performance values of the models trained on patent corpora and the tmChem default model are depicted in Fig. 7.

The F-measure values of all models evaluated on chapati are nearly identical. Additionally, the F-measure scores of the model trained using the chapati corpus are very close on other corpora, perhaps for its small number of instances. Thus, we limit our analysis to the remaining corpora.

The results show that the performance values on the BioS corpus, containing full patents, are demoted when using models trained on CEMP_T or CEMP_D, both of which contain patent abstracts and were annotated using a different annotation guideline. Likewise, the performance values on CEMP_T and CEMP_D are higher when using models trained on CEMPs corpora, and not on BioS. However, we cannot conclude that the models trained on abstracts are not suitable to identify entities from full texts and vice versa, as the corpora are annotated using different annotation guidelines.

The precision, recall and F-measure values achieved in intra-corpus evaluation, shown in Fig. 7, indicate that the precision values on full patents are at least 10% lower than on patent abstracts. Although the recall value on BioS is close to the ones obtained on patent abstracts, its F-measure value is still lower than those of patent abstracts. These results imply that identifying chemical names from full patents is more difficult compared to that of patent abstracts.

## Discussion

We have empirically shown that significant differences exist between the results of chemical NER on patents and scientific articles and even between different types of patent texts. Our study has demonstrated that identifying chemical entities from patent full texts is more complex than from patent abstracts or scientific abstracts. In the following, we assess the complexity of this task on patents, especially on patent full texts.

### Difference between scientific articles and patents

The performance values attained in cross-text-genre evaluations show that the F-measure values of the models trained on the abstracts of scientific articles decrease by around 10% when tested on patent abstracts and by nearly 18% when applied to patent full texts.

The lower F-measure scores obtained by tmChem on patent abstracts compared to that of scientific abstracts, while both have annotation guidelines very similar to those of the tmChem training set, show that there are several chemical entities in patent abstracts that cannot be recognized by the models trained using scientific articles. This finding emphasizes the difficulty of the chemical NER task on patents.

The F-measure scores of ChemSpot trained on scientific abstracts annotated using a guideline different from the ones used for the patent corpora, indicate that these models are not adequate to recognize entities from patents, and accentuate the need for more annotated patent corpora for chemical NER.

#### Execution time analysis

 We compared the execution time of tmChem and ChemSpot over 10 complete patents and 10 journal articles randomly selected from European Patent Office and PubMedCentral, respectively (see “Patent corpora” section). The tools were run single-threaded on a machine with 1TB RAM and Linux operating system. The execution time values, in seconds, are reported in Table 5.

**Table 5:** The execution time, in seconds, of NER tools over 10 full patent documents and 10 journal articles

Text genre	Chemical NER tool	
	ChemSpot	tmChem
10 Patent documents	562	66
10 Scientific articles	*149*	*42*

The execution time values of both systems are lower on scientific articles shown in italic compared with patents

ChemSpot required 149 s to complete the task on scientific articles, around four times faster than the time required for patents. Similarly, tmChem needed approximately 50% more time to finish the task on patents compared with scientific articles. The main reason is the difference in their lengths (see next section).

Then we estimated the execution time of the two systems that one would have to expect on 10 million patents and 10 million full scientific articles, assuming 8 parallel threads by extrapolating the above values. The results show that tmChem would take around 3 months over patents and 2 months over journals while ChemSpot would take approximately 2 years for patents and 7 months for journals. We conclude that large parallel systems are required for patent chemical NER.

#### Text-genre statistics

To better analyze the evident complexity of the NER task on patents apparent from the lower performance and longer execution time of NER tools compared to those of scientific articles, we quantified the differences in their text structures by the average sentence length, document length, and the average number of unique/non-unique TLAs, figures and tables per document (see “Patent corpora” section). We measured the values using 17,000 patent documents and 17,000 journal articles which have been chosen randomly from the European Patent Office and PubMedCentral respectively. The values are provided in Table 6.

**Table 6:** Statistical measurements calculated over 17,000 patent documents and 17,000 journal articles

Text genre	Sentence length	Document length	Number of unique TLAs	Number of TLAs	Number of tables	Number of figures
Patents	21.12	*17,736.00*	*26.75*	*187.47*	*5.34*	*7.10*
Articles	*21.70*	3512.30	8.47	44.73	2.03	2.97

The largest values are represented in italic for each measurement

The average number of words per sentence is almost the same for both patents and journals. However, the average number of words of a patent document is approximately five times higher than that of a journal article, which is in agreement with the findings obtained by Aras et al. [34]. We also observed that the number of TLAs is four times higher in patents than in journal articles, on average. This huge number of TLAs per document makes the NER task on patents harder because of the inherent ambiguity of acronyms. Moreover, the number of tables and figures in patents are more than those in scientific articles. This also makes the extraction of entities from patent documents more difficult than from journal articles [35].

### Difference between patent full texts and patent abstracts

The intra-corpus evaluation scores obtained by retraining tmChem (see “Cross-text-genre to cross-corpus evaluation” section) show that the precision (F-measure) values on abstracts are at least 12% (6%) higher than those on full texts. Since both training and test sets contain documents of the same type (abstracts vs. full texts) annotated with the same annotation guideline, we can conclude that the NER task over patent full texts is more complex than that on patent abstracts.

Moreover, the comparisons at instance level indicate that the patterns of errors observed for FP counts are generally different for different types of patent texts, while they are nearly identical for FN counts. We also infer that filtering just a small number of cases correctly as non-chemicals could reduce the FP or FN values significantly. However, achieving such a filtering is difficult, as shown in the following section.

### Highly ambiguous entities

Results in “Comparison at instance level” section have shown that there are several entities which are frequently observed in both lists of entities with highest FP and FN counts. These are entities whose occurrences can, but need not indicate a chemical depending on their local context in patents. In Tables 7 and 8, we provided full confusion matrices for two entities with this property. The entity “alkyl” is observed on both error lists of the corpora containing patent abstracts, and the entity “H” is found in both error lists of corpora containing full patents. The results show that FP and FN counts are in close proximity for both cases which means that the recognition of the corresponding entities is rather difficult.

**Table 7:** The full confusion matrix for the ambiguous entity “alkyl” calculated for ChemSpot and tmChem over CEMP_T and CEMP_D corpora

ChemSpot CEMP_T	Predicted “alkyl”	Predicted others	tmChem CEMP_T	Predicted “alkyl”	Predicted others
Actual	TP	FN	Actual	TP	FN
“Alkyl”	354	74	“Alkyl”	206	226
Actual	FP	TN	Actual	FP	TN
Others	260	599	Others	39	816

ChemSpot CEMP_D	Predicted “alkyl”	Predicted others	tmChem CEMP_D	Predicted “alkyl”	predicted others
Actual	TP	FN	Actual	TP	FN
“Alkyl”	372	59	“Alkyl”	187	246
Actual	FP	TN	Actual	FP	TN
Others	194	560	Others	37	715

**Table 8:** The full confusion matrix for the ambiguous entity “H” calculated for ChemSpot and tmChem over chapati and BioS corpora containing complete patent documents

ChemSpot chapati	Predicted “H”	Predicted others	tmChem chapati	Predicted “H”	Predicted others
Actual	TP	FN	Actual	TP	FN
“H”	33	37	“H”	36	34
Actual	FP	TN	Actual	FP	TN
Others	11	789	Others	46	754

ChemSpot BioS	Predicted “H”	Predicted others	tmChem BioS	Predicted “H”	Predicted others
Actual	TP	FN	Actual	TP	FN
“H”	344	1869	“H”	309	1921
Actual	FP	TN	Actual	FP	TN
Others	905	135213	Others	3091	133010

### Impact of different annotation guidelines

By comparing the results obtained at the instance level shown in Tables 3 and 4, we noticed that some of the errors are produced due to the differences in the annotation guidelines of NER training sets and patent test sets (see “Patent corpora” section). For example, in these tables, the word “water” is not correctly recognized as a chemical entity by tmChem from BioS corpus or is incorrectly considered chemical by ChemSpot from CEMPs corpora due to the differences in the annotation guidelines.

Moreover, there are many simple chemical elements in the list of entities with high FP counts obtained by tmChem for BioS in Table 3, because simple elements are annotated as chemicals in the training set used for tmChem while they are not labeled as chemicals in BioS corpus. The impact of different rules for annotating simple chemical elements is also observed from the improvement obtained by tmChem in the precision of the BioS corpus after excluding simple chemical elements from both reference and prediction files in “Impact of simple chemical elements” section.

## Conclusion

In this paper, we performed a cross-text-genre evaluation by measuring the tagging quality of the two NER baselines trained on the abstract of scientific articles when evaluated on patent corpora. We noticed that the results are significantly worse on patent corpora compared to scientific abstracts. Although intra-corpus evaluation has shown that training on patent corpora will improve the performance results, performance values are still below the ones achieved for scientific abstracts. Our findings clearly confirm that there are major differences in the NER task between patent and scientific abstracts, and emphasize the complexity of this task on patents.

Moreover, we compared patent abstracts and full texts and addressed the differences between them using various evaluation metrics such as intra-corpus evaluations, and comparison of errors observed at the instance level. We showed that the results on patent abstracts are not extendable to patent full texts which are more important in practice. Therefore, the preparation of more annotated patent full texts is a major requirement for further research in this area.

## Footnotes

1 Abbreviated as “chemical” from now on.

2
http://www.ncbi.nlm.nih.gov/pmc.

3 See http://www.biocreative.org.

4 See https://www.epo.org/.

5 See https://www.ebi.ac.uk/chebi/.

6 See http://www.biosemantics.org/.

7 TLA is defined as any three-letter word with letters all in uppercase form.

8 See https://www.epo.org/.

9 Although the performance of ChER was very close to that of tmChem in the BioCreative IV challenge, we were not able to include it in our study as it is not freely available.

10 Note that ChemSpot was not evaluated in the BioCreative IV challenge.

11 See http://www.cnts.ua.ac.be/conll2000/chunking.

12 In this format, every non-letter and non-digit character, and all number-letter changes are split.

13 Version (stanford-parser 3.5) is available in http://nlp.stanford.edu/software/lex-parser.shtml.

14 See http://www.chemicalelements.com.
